# USP44 accelerates the growth of T-cell acute lymphoblastic leukemia through interacting with WDR5 and repressing its ubiquitination

**DOI:** 10.7150/ijms.74535

**Published:** 2022-11-14

**Authors:** Zuofei Chi, Bin Zhang, Ruowen Sun, Ye Wang, Linlin Zhang, Gang Xu

**Affiliations:** The Second Department of Pediatric Hematology, Shengjing Hospital of China Medical University, Shenyang 110004, Liaoning, China.

**Keywords:** T-cell acute lymphoblastic leukemia, USP44, WDR5, ubiquitination

## Abstract

T-cell acute lymphoblastic leukemia (T-ALL) is a common hematologic malignancy. Based on the data from GSE66638 and GSE141140, T-ALL patients depicted a higher USP44 level. However, its role in T-ALL is still unclear. In the present study, we investigated the role of USP44 in T-ALL growth. USP44 overexpression elevated the proliferation of CCRF-CEM cells, while USP44 knockdown suppressed the proliferation of Jurkat and MOLT-4 cells. In addition, USP44 accelerated the cell cycle progression, with boosted cyclinD and PCNA levels. However, USP44 knockdown induced apoptosis in Jurkat and MOLT-4 cells, with an upheaval among cleaved caspase-3 and PARP levels. Mechanistically, USP44 co-localized and interacted with WDR5, leading to the repression of its ubiquitination and degradation. Interestingly, WDR5 overexpression abolished the apoptosis induced by USP44 knockdown. Consistently, the* in vivo* study revealed that USP44 knockdown restricted the leukemic engraftments in the bone marrow and spleens and reduced the infiltration of T-ALL cells in the livers and lungs. In conclusion, this study indicated that USP44 enhanced the growth of T-ALL through interacting with WDR5 and repressing its ubiquitination. This study highlights the potential use of USP44 as a therapeutic target of T-ALL.

## Introduction

T-cell acute lymphoblastic leukemia (T-ALL), a common malignancy in the hematologic system, is arisen from genetic lesion accumulation during T-cell development, leading to the differentiation arrest and abnormal proliferation of immature progenitors. During T-ALL, leukocytosis initiates and infiltrates within the lymph nodes and other organs 1. T-ALL accounts for 15% of pediatric and 25% of adult ALL patients 1. So far, chemotherapy is the primary treatment for T-ALL. However, high-intensity chemotherapy will result in short-term and long-term side effects, lacking effective targeted therapy. Therefore, it is urgent to understand the molecular pathogenesis of T-ALL.

Ubiquitination, a form of post-translation modification, leads to protein degradation. The ubiquitination/deubiquitination system is critical in various physiological events by participating in intracellular signaling pathways 2, 3. Ubiquitin-specific protease 44 (USP44), a kind of deubiquitinase, is implicated in modulating innate immune responses 4, spindle checkpoints and centrosome positioning 5, stem cell differentiation 6 and DNA damage response 7. Recently, several studies revealed the characteristics of USP44 in cancers. USP44 is highly expressed in gastric cancer and glioma 8, 9. In contrast, it is poorly expressed in lung, colon, breast, renal and pancreatic cancers 10-14, and is implicated in their growth and metastasis. Interestingly, Ying *et al.* demonstrated a high USP44 level in T-ALL 15, with little knowledge about its role.

The WD repeat protein family members regulate various cellular events and are considered as potential targets for disease treatment 16. WD repeat domain 5 (WDR5) is a core component of the histone H3 lysine 4 (H3K4) methyltransferase complex, which catalyzes histone H3K4 tri-methylation (H3K4me3) 17. H3K4me3 is associated with the transcription activation of target genes 18, 19 and regulates the expression of genes associated with protein synthesis 20. On the other hand, WDR5 is also a crucial co-activator for recruiting transcription factors, including c-Myc, to chromatin and transcription activation 21, 22. It is implicated in multiple biological processes, such as tumorigenesis, vertebrate development, and self-renewal. WDR5 is highly expressed in various cancer cells and is associated with clinicopathological parameters and poor prognoses. The WDR5 expression in T-ALL patients is also higher than normal controls and is related to the increased risk of leukemia 23. Knockdown of WDR5 suppresses the proliferation of leukemia cells 23. Based on data from the HitPredict Database (http://www.hitpredict.org/), WDR5 may bind with USP44. However, whether WDR5 mediates the functions of USP44 is unknown.

In this study, the role of USP44 in T-ALL was explored. It was revealed that USP44 accelerated the growth of T-ALL cells by interacting with WDR5 and suppressing its ubiquitination and degradation. This study depicted a potential role of USP44 as a candidate target for treating T-ALL.

## Materials and Methods

### Gene expression of USP44

GEO DataSets (GSE66638 and GSE141140) were utilized to analyze the expression of USP44 within T-ALL samples and normal cells.

### Cell culture

Human T-ALL cell lines Jurkat, MOLT-4 and CCRF-CEM were procured from iCell Bioscience Co., Ltd (Shanghai, China) and cultured in RPMI-1640 medium (Gibco Biotechnology Co., Ltd, Grand Island, USA) supplemented with 10% fetal bovine serum (FBS; Biological Industries, Cromwell, USA). Human HEK-293T cells were procured from ZhongQiaoXinZhou Biotechnology Co., Ltd (Shanghai, China) and grown in DMEM medium (Servicebio Biotechnology Co., Ltd, Wuhan, China) with 10% FBS. All the cells were cultured under a humidified atmosphere with 5% CO_2_ at 37 °C.

### Infection and transfection

USP44 CDS was inserted into a pLVX-IRES-puro plasmid (Fenghui Biotechnology Co., Ltd, Hunan, China) and infected into CCRE-CEM cells through a lentiviral infection system. Blank pLVX-IRES-puro plasmid served as the negative control. USP44 shRNAs were inserted into a pLVX-shRNA1 plasmid (Fenghui Biotechnology Co., Ltd) and infected into Jurkat and MOLT-4 cells through a lentiviral infection system. No-targeting sequence inserted into a pLVX-shRNA1 plasmid served as the negative control. The sequence information was as follows:

- LV-shUSP44-1#: CCGGCTTCAAAGTGAAGATCAACTTTCAAGAGAAGTTGATCTTCACTTTGAAGCTTTTT;
- LV-shUSP44-2#: CCGGGCACTGTGTGGACTGCAACATTCAAGAGATGTTGCAGTCCACACAGTGCCTTTTT;
- LV-shNC: CCGTTCTCCGAACGTGTCACGTTTCAAGAGAACGTGACACGTTCGGAGAATTTTT.

The cells were selected using puromycin (2-2.5 μg/ml; Aladdin Biotechnology Co., Ltd, Shanghai, China). HA-Ubiquitin, Flag-WDR5, and His-USP44 overexpression plasmids (GeneScript Biotechnology Co., Ltd, Nanjing, China) were transfected into HEK-293T cells, and WDR5 overexpression was transfected into Jurkat cells using Effectene Transfection Reagent (Qiagen Biotechnology Co., Ltd, Germantown, USA) according to the manufacturer's protocol. Finally, the cells were collected for subsequent experiments after 48h.

### Quantitative real-time PCR

Total mRNA was extracted from the cells using TRIpure Reagent (BioTeke Biotechnology Co., Ltd, Beijing, China). After cDNA synthesis using BeyoRT II M-MLV (Beyotime Biotechnology Co., Ltd, Shanghai, China) based on the instruction, quantitative real-time PCR was carried out using 2×Taq PCR MasterMix (Solarbio Biotechnology Co., Ltd, Beijing, China) and SYBR Green (Solarbio Biotechnology Co., Ltd) on an Exicycler 96 PCR system (BIONEER, Daejeon, Korea). CDNA served as the template. The following primers were used: forward primer for USP44, 5'-CAA CTT ATG ATA TGC CAC CTA-3'; reverse primer for USP44, 5'-GTA CCC AGA ACC CTC CT-3'; forward primer for WDR5, 5'-CAC CTG TGA AGC CAA ACT-3'; reverse primer for WDR5, 5'-GAG GCA GAA ACA AGA AGG-3'; forward primer for GAPDH, 5'-GAC CTG ACC TGC CGT CTA G-3'; reverse primer for GAPDH, 5'-AGG AGT GGG TGT CGC TGT-3'. The thermal circulation is as follows: 94 °C for 5 min; 94 °C for 10 s, 60 °C for 20 s, and 72 °C for 30 s, 40 cycles; then 72 °C for 2.5 min and 40 °C for 1.5 min. It was melting form 60 °C to 94 °C, 1 °C/1 s. Gene expression was determined based on the 2^-ΔΔCT^ method and normalized to GAPDH.

### CCK-8

After infection or transfection for 48 h, the cells were seeded into 96-well plates (4×10^3^/well). According to the instruction, the cell viability was assessed using CCK-8 reagent (KeyGen Biotechnology Co., Ltd, Nanjing, China) at indicated time points. Absorbance at 450 nm was measured with a microplate reader (BIOTEK, Winooski, USA).

### Western blot and co-immunoprecipitation

For western blot, the cells were lysed using RIPA with 1 mM protease inhibitor PMSF (Solarbio Biotechnology Co., Ltd). The supernatants were harvested through centrifugation at 10000 xg for 5 min at 4 °C. After measuring the protein concentration using a BCA protein assay kit (Solarbio Biotechnology Co., Ltd), 20 μg of whole cell lysates were subjected to SDS-PAGE, followed by electroblotting onto the polyvinylidene fluoride membranes (Millipore Biotechnology Co., Ltd, Bedford, USA). After blockade using 5% skim milk and washing using TBST buffer, the membranes were incubated with primary antibodies overnight at 4 °C. It was followed by washing using TBST buffer and incubating with corresponding secondary antibodies (1: 3000; Solarbio Technology Co., Ltd) at 37°C for 1 h. Then the targeted proteins were washed using TBST buffer and visualized by enhanced chemiluminescence (Solarbio Biotechnology Co., Ltd). The following primary antibodies were utilized: anti-USP44 (1: 300; Santa Cruz Biotechnology Co., Ltd, Dallas, USA), anti-Cyclin D1 (1: 1000; Cell Signaling Technology Co., Ltd, Beverly, USA), anti-PCNA (1: 1000; Cell Signaling Technology Co., Ltd), anti-cleaved caspase-3 (1: 1000; Cell Signaling Technology Co., Ltd), anti-cleaved PARP (1: 1000; Cell Signaling Technology Co., Ltd), anti-WDR5 (1: 300; Santa Cruz Biotechnology Co., Ltd), and anti-GAPDH (1: 2000; Cell Signaling Technology Co., Ltd). The densitometric quantification of blots was undergone using the Gel-Pro-Analyzer software.

Based on the instruction, the cell lysates were incubated with bead-USP44 or bead-WDR5 for immunoprecipitation after measurement of protein concentration. The immunoprecipitates were washed with PBS, eluted with 50 μl elution buffer and subjected to western blot as described above. The antibodies used for the western blot were USP44 (1: 300) and WDR5 (1: 300).

### Ubiquitination assay

HA-Ubiquitin, Flag-WDR5, and His-USP44 were co-transfected into HEK-293T cells. After 48 h, the cells were treated using 5 μM MG132 (Aladdin Biotechnology Co., Ltd) for 6 h to repress the proteasomal degradation. After that, the cells were harvested, and the ubiquitination status of WDR5 was determined through immunoprecipitation, as description above. Briefly, the whole cell lysates were immunoprecipitated with bead-flag and subjected to western blot. The antibodies utilized in western blot were flag (1: 3000, Abcam Technology Co., Ltd, Cambridge, UK), his (1: 1000; Beyotime Biotechnology Co., Ltd, Shanghai, China) and HA (1: 1000; Beyotime Biotechnology Co., Ltd).

### Flow cytometry analysis

For cell cycle determination, the cells were incubated with 10 μM BrdU (Aladdin Biotechnology Co., Ltd) for 40 min after indicated treatment and then harvested, fixed, permeabilized and labeled using a FITC-BrdU determination kit (KeyGen Biotechnology Co., Ltd) and a 7-ADD cell cycle determination kit (Bestbio, Changsha, China) based on the protocol. After that, the cells were analyzed through a flow cytometer (NovoCyte, Agilent Technologies, Inc., Santa Clara, USA). For determinating cell apoptosis, the cells were harvested, washed using PBS and stained with a cell apoptosis determination kit (KeyGen Biotechnology Co., Ltd) following the protocol. Thereafter, the cell apoptosis was determined with a flow cytometer.

The femurs and tibias of mice were harvested to obtain the bone marrow. The spleen tissues of mice were also harvested. Cells in the bone marrow and spleens were incubated with CD45 primary antibody (MultiSciences Biotechnology Co., Ltd, Hangzhou, China) at 4 °C for 30 min. Later, the cells were analyzed with a flow cytometry.

### Immunofluorescence

Cells were fixed in 4% paraformaldehyde, permeabilized in 0.1% TritonX-100 and blocked using 1% BSA. Thereafter, the cells were incubated with antibodies against WDR5 (1: 50; Santa Cruz Biotechnology Co., Ltd) or USP44 (1: 100; Affinity, Changzhou, China) at 4 °C overnight, followed by incubating with Cy3-conjugated (1: 200; Invitrogen Biotechnology Co., Ltd, Carlsbad, USA) or FITC-conjugated secondary antibodies (1: 200; Abcam Technology Co., Ltd) at room temperature for 60 min. DAPI (Aladdin Biotechnology Co., Ltd, Shanghai, China) was incorporated to stain the nuclei. After washing with PBS, cell images were visualized using a fluorescence microscope (Olympus, Tokyo, Japan). The co-localization of USP44 and WDR5 was analyzed with Image Pro Plus software.

### Protein half-life assay

In order to determine the half-time of WDR5 in Jurkat cells with USP44 knockdown, the cells were treated with 10 μg/ml CHX (Aladdin Biotechnology Co., Ltd) to block protein translation. After treatment with CHX for 0, 2, 4, and 8 h, the cells were collected, and western blot was conducted to determine the WDR5 protein level.

### Animal experiment protocol

Six-week-old NOD-SCID mice were procured from Beijing Huafukang Biotechnology Co., Ltd (Beijing, China) and fed within a standard condition. USP44 knockdown cells (LV-shUSP44-1#) were injected into mice through the caudal vein (2×10^5^). At the end of the study, mice were sacrificed. The bone marrow and spleens were obtained to determine CD45^+^ cells. The images and weight of spleens were recorded. The lung and liver tissues were harvested for hematoxylin and eosin (HE) and immunohistochemistry (IHC) staining using CD3. All the experiments were conducted following the Guide for the Care and Use of Laboratory Animals and were approved by the Institutional Ethics Committee of Shengjing Hospital of China Medical University.

### IHC

The lung and liver tissues were harvested, fixed in 4 % paraformaldehyde, embedded in paraffin, and cut into 5-μm slices. After deparaffinage, rehydration and antigen retrieval, the slices were treated with 3% H_2_O_2_ to inactivate endogenous peroxidase, and then blocked with 1% BSA. Then, the slices were incubated with CD3 primary antibody (1: 100; Abclonal Biotechnology Co., Ltd, Wuhan, China) at 4°C overnight. It was followed by incubation with peroxidase-conjugated secondary antibodies (1: 500; ThermoFisher Scientific Technology Co., Ltd, Waltham, USA) for 60 min at 37 °C. The slices were visualized using DAB (MXB Biotechnology, Fuzhou, China) and counterstained with hematoxylin (Solarbio Biotechnology Co., Ltd). The images were captured under a microscope.

### Statistical analysis

All the results were represented as mean and SD. Comparison between the groups was analyzed using Student's *t* test or one-way analysis of variance followed by Tukey's post hoc test. A *P-*value < 0.05 was considered statistically significant.

## Results

### USP44 was highly expressed in T-ALL patients

**Figure 1:**
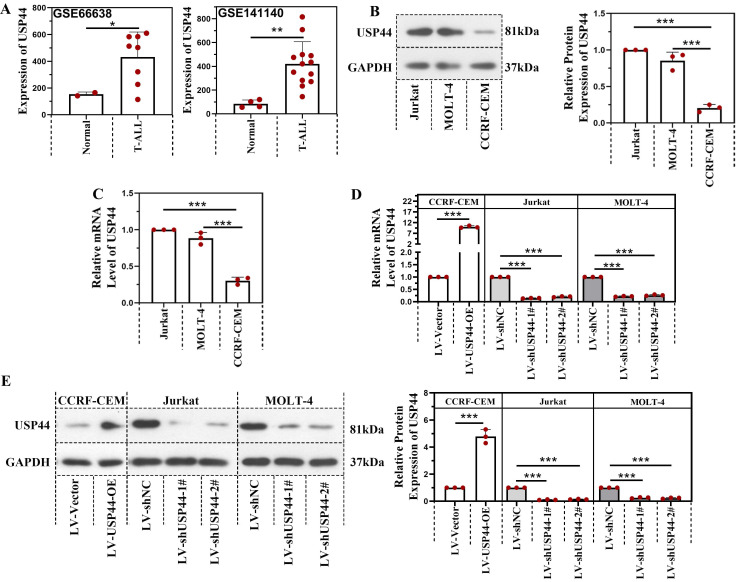
** USP44 was highly expressed in T-ALL patients.** (**A**) The level of USP44 among human T-ALL samples and normal cells based on the data from GSE66638 and GSE141140. (**B**) The protein level of USP44 in human T-ALL cell lines Jurkat, MOLT-4, and CCRF-CEM, was detected using western blot. (**C**) The mRNA level of USP44 in T-ALL cell lines Jurkat, MOLT-4, and CCRF-CEM was determined through quantitative real-time PCR. (**D**) USP44 shRNA (LV-shUSP44-1# and LV-shUSP44-2#) or USP44 overexpression (LV-USP44-OE) was infected into cells through the lentivirus system. After that, the USP44 mRNA level was determined using quantitative real-time PCR. (**E**) The protein level of USP44 in cells infected with USP44 shRNA or overexpression was determined using western blot. All the results were represented as mean and SD. * p< 0.05, *** p< 0.001.

Based on the data from GSE66638 and GSE141140, the USP44 level in T-ALL patients was higher than in normal cells (Figure 1A). The USP44 level in Jurkat, MOLT-4, and CCRF-CEM cells were determined using quantitative real-time PCR and western blot to further explore the role of USP44 in T-ALL. As shown in Figure 1B-C, Jurkat and MOLT-4 cells revealed a higher USP44 level than the CCRF-CEM cells. Thus USP44 in Jurkat and MOLT-4 cells were knockdown, and USP44 was overexpressed in CCRF-CEM cells through the lentivirus infection system. Thereafter, the infection efficiency was detected by quantitative real-time PCR and western blot. In CCRF-CEM cells, the overexpression of USP44 elevated the USP44 level, while in Jurkat and MOLT-4 cells, knockdown of USP44 decreased the level of USP44 significantly, at both mRNA and protein levels (Figure 1D-E).

### USP44 accelerated the proliferation and cell cycle progression of T-ALL cells

**Figure 2:**
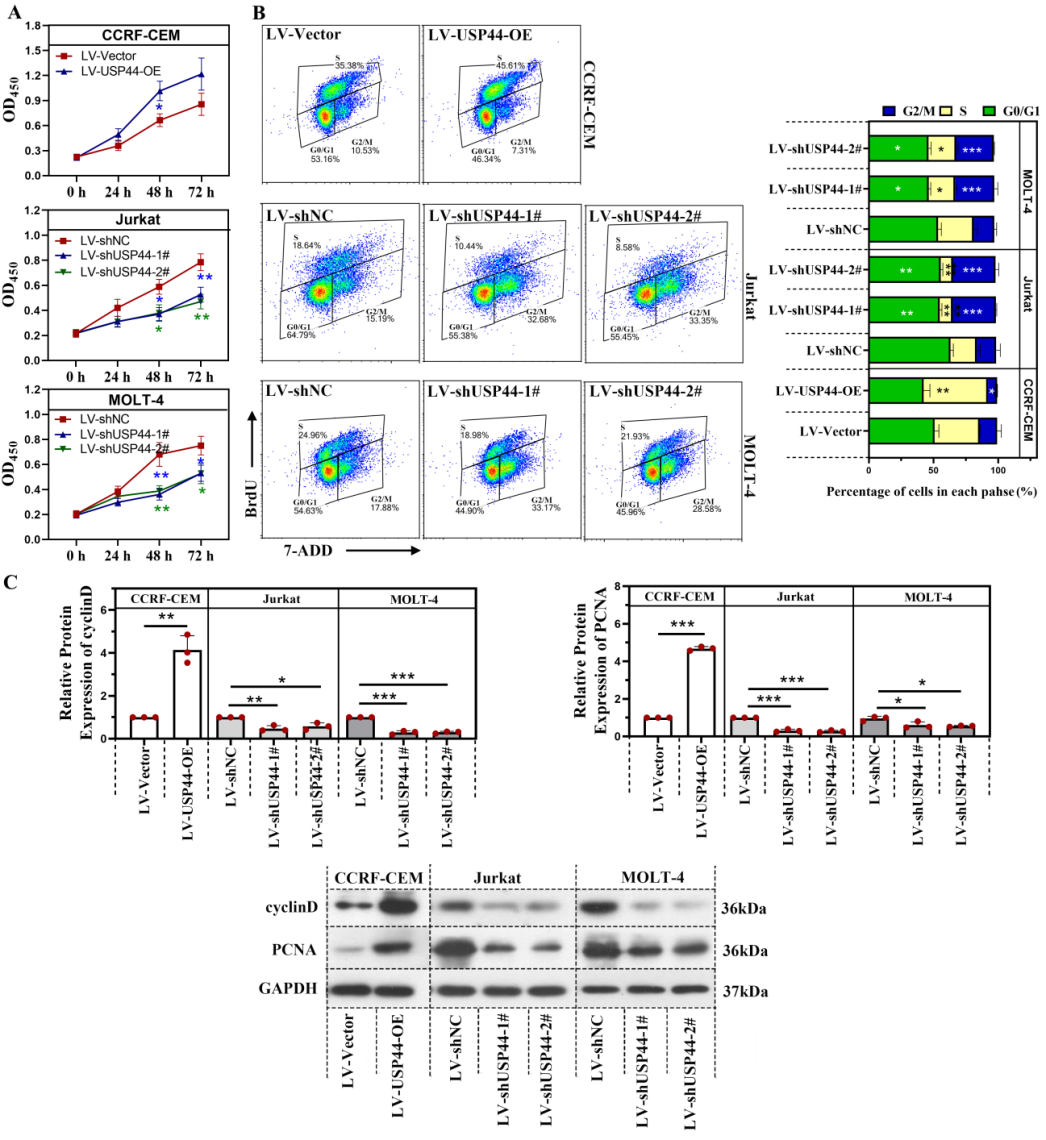
** USP44 accelerated the proliferation and cell cycle progression of T-ALL cells.** (**A**) The proliferation of CCRF-CEM cells with USP44 overexpression (LV-USP44-OE) and Jurkat and MOLT-4 cells with USP44 knockdown (LV-shUSP44-1# and LV-shUSP44-2#) was assessed by CCK-8 assay. (**B**) Cell cycle of cells with/without USP44 overexpression or knockdown was determined using a flow cytometry analysis. (**C**) The protein levels of cyclinD and PCNA in cells with USP44 overexpression or knockdown were evaluated by western blot. All the results were presented as mean and SD. * p< 0.05, ** p< 0.01, *** p< 0.001.

First, we explored the role of USP44 in the proliferation of T-ALL cells. Overexpression of USP44 promoted the proliferation of CCRF-CEM cells. However, the knockdown of USP44 impeded the proliferation of Jurkat and MOLT-4 cells (Figure 2A). To further explore the role of USP44 in cell cycle progression, the cell cycle of T-ALL cells with USP44 overexpression or knockdown was detected with flow cytometry. In cells with USP44 overexpression, the percentages of cells in G2/M phases decreased, while the percentage of cells in S phase increased significantly (Figure 2B). In cells with USP44 knockdown, the percentage of cells in G2/M phase increased, while those in G0/G1 phase and S phase decreased significantly (Figure 2B). On the other hand, the protein levels cyclinD and PCNA among cells with USP44 overexpression or knockdown were also determined through western blot. In cells with USP44 overexpression, the levels of PCNA and cyclinD were elevated. In contrast, in cells with USP44 knockdown, the levels of PCNA and cyclinD were reduced (Figure 2C). These results indicated that USP44 could promote the proliferation and accelerate the cell cycle progression of T-ALL cells.

### Knockdown of USP44 induced the apoptosis of T-ALL cells

**Figure 3:**
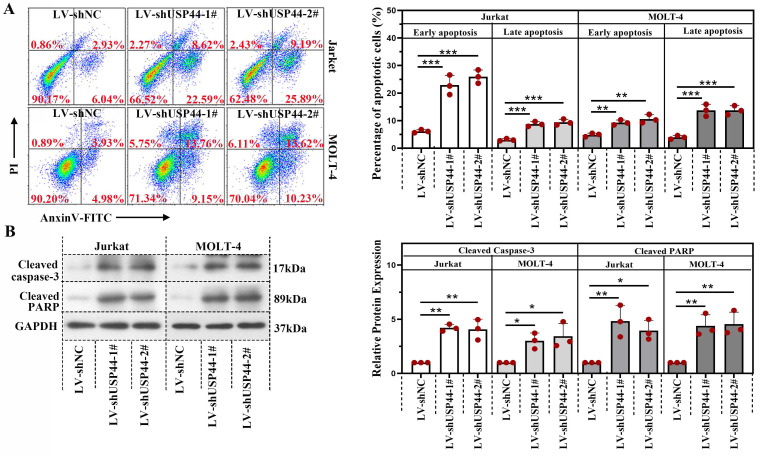
** Knockdown of USP44 induced apoptosis of T-ALL cells.** (**A**) Apoptosis in Jurkat and MOLT-4 cells with/without UPS44 knockdown (LV-shUSP44-1# and LV-shUSP44-2#) was analyzed using a flow cytometry. (**B**) The protein levels of cleaved caspase-3 and cleaved PARP were determined by western blot. All the results were represented as mean and SD. * p< 0.05, ** p< 0.01, *** p<0.001.

We explored the role of USP44 in the apoptosis of T-ALL cells. In cells having USP44 knockdown, the total percentage of apoptotic cells was elevated significantly, with both early apoptosis and late apoptosis (Figure 3A). Meanwhile, the cleaved caspase-3 and cleaved PARP levels were also increased (Figure 3B). These results depicted that the knockdown of USP44 induced the apoptosis of T-ALL cells.

### USP44 interacted with WDR5 and reduced its degradation mediated by ubiquitination

**Figure 4:**
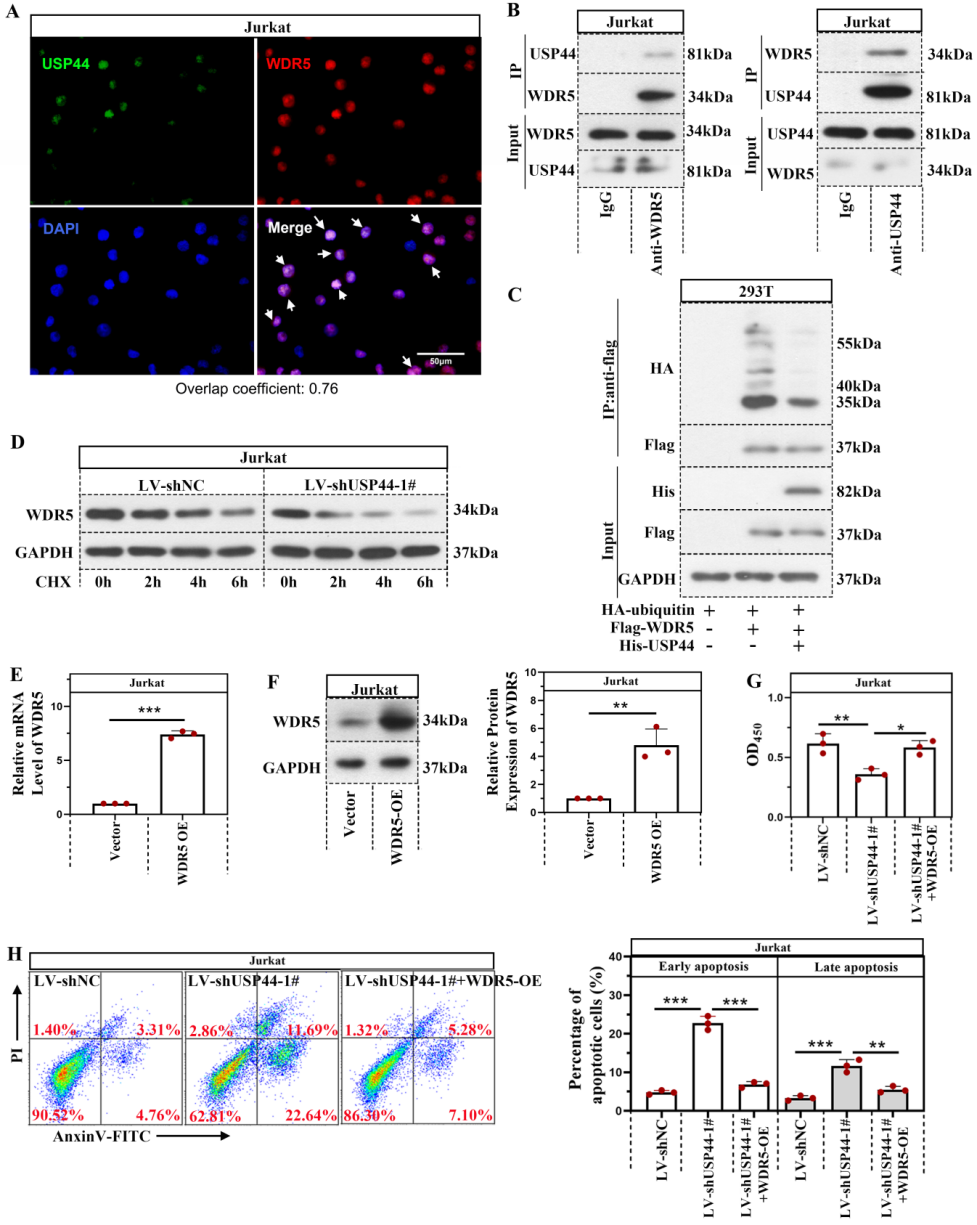
** USP44 interacted with WDR5 and reduced its degradation through ubiquitination.** (**A**) Immunofluorescence staining using anti-USP44 and anti-WDR5 was conducted. (**B**) Co-immunoprecipitation was conducted with anti-WDR5 or anti-USP44 antibodies to verify the binding between USP44 and WDR5. IgG served as the negative control. (**C**) Among HEK-293T cells transfected with HA-ubiquitin, flag-WDR5, and/or his-USP44, the ubiquitination level of WDR5 was detected through co-immunoprecipitation with an anti-flag antibody. (**D**) Protein degradation of WDR5 in cells with USP44 silencing was detected using western blot after treatment with CHX. (**E**) The relative mRNA level of WDR5 after transfection with WDR5 overexpression was determined by quantitative real-time PCR. (**F**) Western blot was conducted to validate the transfection efficiency of WDR5 overexpression. (**G**) The viability of cells with USP44 knockdown (LV-shUSP44) and WDR5 overexpression (WDR5-OE) was determined using CCK-8 assay. (**H**) Flow cytometry was conducted to determine the apoptosis of cells with USP44 knockdown and WDR5 overexpression. All the results were represented as mean and SD. * p< 0.05, ** p< 0.01, *** p<0.001.

Immunofluorescence staining demonstrated that USP44 was co-located with WDR5 (Overlap coefficient=0.76; Figure 4A). USP44 also interacted with WDR5, evidenced by co-immunoprecipitation (Figure 4B). As USP44 is a deubiquitinase, we also described the influence of USP44 on the ubiquitination of WDR5. Compared with cells transfected without His-USP44, the ubiquitination level of WDR5 among cells transfected with His-USP44 was reduced (Figure 4C). It indicated that USP44 interacted with WDR5 and decreased its ubiquitination. As ubiquitination is an important event during protein degradation, we also detected the degradation of WDR5. WDR5 degraded more rapidly in cells with USP44 knockdown when compared with negative control cells (Figure 4D). We wondered whether USP44 performed its function by targeting WDR5. Thus a WDR5 overexpression was transfected into cells with USP44 knockdown. The efficiency of WDR5 overexpression was evidenced through quantitative real-time PCR and western blot (Figure 4E-F). Interestingly, the cell viability, inhibited by USP44 knockdown, was elevated after WDR5 overexpression (Figure 4G). Moreover, the cell apoptosis, induced by USP44 knockdown, was suppressed by WDR5 overexpression (Figure 4H). These data demonstrated that USP44 might promote the growth of T-ALL cells through interacting with WDR5 and reducing its degradation mediated by ubiquitination.

### USP44 knockdown repressed the T-ALL progression* in vivo*

**Figure 5:**
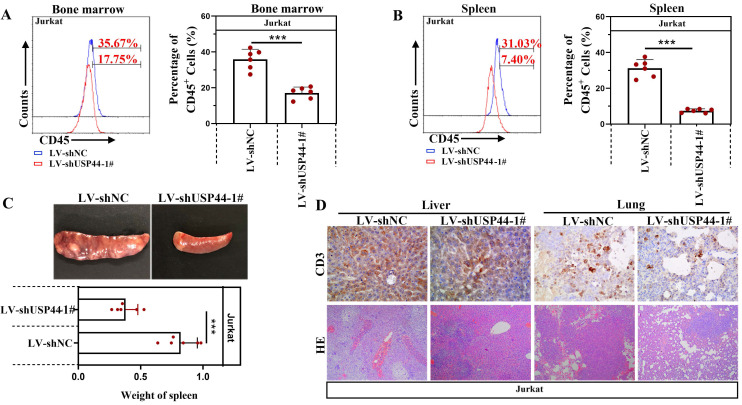
** USP44 knockdown repressed the progression of T-ALL *in vivo.*** (**A**) CD45^+^ T-ALL cells inside the bone marrow of mice injected with cells with/without USP44 knockdown (LV-shUSP44) were detected using flow cytometry. (**B**) CD45^+^ T-ALL cells inside the spleens of mice injected with cells with/without USP44 knockdown were analyzed with flow cytometry. (**C**) Images of spleens and spleen weights of mice in each group. (**D**) HE staining (100x) and IHC staining with CD3 (400x) in the livers and lungs of mice in each group. Typical images were represented. All the results were represented as mean and SD. N=6. *** p<0.001.

Jurkat cells with/without USP44 knockdown were injected into NOD-SCID mice to verify the function of USP44 on the progression of T-ALL* in vivo*. CD45 indicated leukemic cells in the bone marrow and spleens. Compared with the negative control (LV-shNC), the percentage of CD45^+^ cells in the bone marrow decreased when USP44 was knockdown (Figure 5A). Also, in mice injected with USP44 knockdown cells, the percentage of CD45^+^ cells in the spleens was reduced (Figure 5B). These results demonstrated that USP44 knockdown declined leukemic engraftments in the bone marrow and spleens. In addition, the spleens of mice injected with USP44 knockdown cells were much smaller, with a lower spleen weight than those of negative control mice (Figure 5C). On the other hand, T-lymphocytes were indicated by CD3. The results of HE and IHC staining with anti-CD3 showed that fewer T-ALL cells were infiltrated into the livers and lungs of mice injected with USP44 knockdown cells than the negative control mice (Figure 5D). These results supported our hypothesis that knockdown of USP44 repressed the T-ALL progression *in vivo*.

## Discussion

Several USPs are involved in leukemia. For instance, USP15 deletion injures leukemic progenitor function and derepresses antioxidant responses through the Keap1-Nrf2 pathway 24. Targeting USP47 can overcome the resistance to tyrosine kinase inhibitors and eradicate leukemia progenitor cells 25. USP15 safeguards the hematopoiesis and genome integrity within hematopoietic stem cells 26. In our study, USP44 was highly expressed in T-ALL patients and accelerated the proliferation of T-ALL cells, indicating that USP44 could become a candidate target for treating T-ALL. Consistently, USP44 also aggravates the malignancy of glioma and tumorigenesis of prostatic cancer cells 8, 27. Interestingly, the function of USP44 in tumorigenesis could be tumor-dependent. In several cancers, including colon, breast, pancreatic, renal, and non-small cell lung cancer, USP44 depicted a low expression. USP44 suppressed the growth and metastasis, and induced apoptosis 10-14. The level of USP44 is also associated with tumor grades, survival and poor prognosis 10, 13. Unfortunately, due to a lack of clinical sample information, the association between USP44 and T-ALL stages, survival, and prognosis remains unclear and requires further exploration.

In our study, USP44 contributed to the growth of T-ALL cells. As the cell cycle is a critical regulator of cell growth, we also investigated the role of USP44 in the cell cycle modulation and depicted that USP44 accelerated cell cycle progression. Noticeably, overexpression of USP44 increased the percentage of cells in S phase, in which phase DNA synthesis is processed, indicating that USP44 might promote the synthesis of DNA. In addition, we showed that knockdown of USP44 increased the percentage of cells in G2/M phase. USP44 has a role in mitosis regulation 28, which is an integral part of the cell cycle. Interestingly, USP44 altered mitotic arrest kinetics, leading to a reinforced mitotic checkpoint 15. Thus, we hypothesized that USP44 knockdown may suppress the mitosis of T-ALL cells, contributing to its role in the growth of T-ALL cells. Consistently, USP44 increases the level of cyclinB 15, which participates in the transition checkpoint from G2 to M phase. Moreover, we revealed that USP44 also up-regulated the level of cyclinD, which participates in the transition checkpoint from G1 to S phase. This is consistent with our speculation that USP44 could accelerate DNA synthesis.

USP44 also regulated apoptosis of cancer cells in a cell-dependent way. Interestingly, USP44 induced the apoptosis of colon cancer cells 11, while suppressed the apoptosis of glioma 8. In our study, we demonstrated that USP44 knockdown elevated the apoptosis of T-ALL cells, revealing a suppression role of USP44 during the apoptosis of T-ALL cells. Meanwhile, the enhanced apoptosis induced by USP44 knockdown was also accompanied by increased caspase-3 and PARP activation, which are essential effectors in apoptotic pathways.

WDR5, an oncogene often found in cancers, is associated with the proliferation, cell cycle, and apoptosis of cancer cells 29-32. WDR5 could regulate the expression of cyclinD and DNA damage by H3K4me3 33, 34. The suppression of WDR5 signal can repress the growth of cancer cells 34. WDR5 also has a critical role in leukemogenesis 17. Our study observed that USP44 bound with WDR5, while silencing USP44 accelerated WDR5 degradation, indicating that USP44 bound with WDR5 and regulated its stability. Additionally, USP44 decreased the ubiquitination of WDR5, which could contribute to its function during WDR5 degradation. Furthermore, WDR5 overexpression abolished the effects of USP44 on the proliferation and apoptosis of T-ALL cells, revealing that USP44 functioned in T-ALL cells by interacting with WDR5, thereby suppressing the ubiquitination and degradation of WDR5. Interestingly, the WDR5 degrader inhibits the proliferation of acute myeloid leukemia and reduces the level of chromatin-bound c-myc, which is critical in T-ALL pathogenesis 35. Hence, we wonder whether USP44 affects the interaction between WDR5 and c-myc, and this needs further exploration in our further study. Other signaling pathways, such as the AKT and β-catenin signals 10, 11, 30, 32, were also modulated by WDR5. These signal pathways may also participate in the function of USP44 in T-ALL. However, more explorations are needed to reveal the underlying mechanisms behind USP44 in T-ALL.

Besides, USP44 and WDR5 are also associated with the metastasis of cancer, such as the migration and invasion capability of cancer cells and vasculogenic mimicry 12, 13, 27, 36-38. WDR5 is also related to the liver infiltration of leukemia cells 23. In our study, when USP44 was knockdown, the level of leukemia cells in the bone marrow and spleens was reduced, and the level of leukemia cells infiltrated into the livers and lungs also declined. It indicated that USP44 promotes both the growth and infiltration of leukemia cells. Recently, programmed death-ligand 1 (PD-L1), which is associated with immune escape of cancer cells 39, has caught the attention of researchers. WDR5 was reported to promote the expression of PD-L1 in prostate cancer and associated with its radio- and chemo- resistance 34, 40. We speculated that USP44 could also be associated with the immune escapes of T-ALL cells, which needs further investigation.

Collectively, in the present study, USP44 contributed to the growth of leukemia cells and suppressed their apoptosis. It also regulated the progression of T-ALL *in vivo* and their infiltration in the livers and lungs. Significant research demonstrated that USP44 interacted with WDR5 and repressed its ubiquitination and degradation, contributing to the role of USP44 in T-ALL. Our results depicted that USP44 could become a potential therapeutic target for treating T-ALL.
